# Activating Death Receptor DR5 as a Therapeutic Strategy for Rhabdomyosarcoma

**DOI:** 10.5402/2012/395952

**Published:** 2012-04-17

**Authors:** Zhigang Kang, Shi-Yong Sun, Liang Cao

**Affiliations:** ^1^Genetics Branch, Center for Cancer Research, National Cancer Institute, Bethesda, MD 20892, USA; ^2^Laboratory of Proteomics and Analytical Technologies, SAIC-Frederick, Inc., NCI Frederick, Frederick, MD 21702, USA; ^2^Department of Hematology and Medical Oncology, Winship Cancer Institute, Emory University School of Medicine, Atlanta, GA 30322, USA

## Abstract

Rhabdomyosarcoma (RMS) is the most common soft tissue sarcoma in children. It is believed to arise from skeletal muscle progenitors, preserving the expression of genes critical for embryonic myogenic development such as *MYOD1* and *myogenin*. RMS is classified as embryonal, which is more common in younger children, or alveolar, which is more prevalent in elder children and adults. Despite aggressive management including surgery, radiation, and chemotherapy, the outcome for children with metastatic RMS is dismal, and the prognosis has remained unchanged for decades. Apoptosis is a highly regulated process critical for embryonic development and tissue and organ homeostasis. Like other types of cancers, RMS develops by evading intrinsic apoptosis via mutations in the *p53* tumor suppressor gene. However, the ability to induce apoptosis via the death receptor-dependent extrinsic pathway remains largely intact in tumors with *p53* mutations. This paper focuses on activating extrinsic apoptosis as a therapeutic strategy for RMS by targeting the death receptor DR5 with a recombinant TRAIL ligand or agonistic antibodies directed against DR5.

## 1. Introduction

Rhabdomyosarcoma (RMS) is the most common pediatric soft-tissue tumor. Despite extensive research and aggressive clinical management, the overall outcome for children with metastatic disease is dismal with a prognosis largely unchanged in decades [1, 2]. RMS tumors are histologically classified into two major subtypes, embryonic (ERMS) and alveolar (ARMS), which are associated with unique genetic changes. The majority of ARMSs are characterized by the presence of P*AX3/7:FOXO1 *translocation [3, 4]. ERMSs, on the other hand, are more frequently associated with activated RAS signaling via mutations in *RAS* genes or deletions in *NF1*, a tumor suppressor that encodes an RAS inhibitor [5–7].

The two subtypes of RMS also have distinct prognoses. ERMSs are often found in younger patients who generally do better, whereas ARMSs are more frequently diagnosed in adolescents and young adults who have a worse prognosis with a five-year survival rate of less than 50% [8–11]. Additional mutations in tumor suppressors are important for the development of RMS. In particular, RMS is the most common pediatric cancer in families with Li-Fraumeni syndrome [12]. Mutations in *p53* are important for pathogenesis and commonly found in RMS [13, 14].

Despite advances in radiation and chemotherapy, there has been little change in the 5-year survival rate for pediatric RMS [10]. The cure rate for advanced RMS is not expected to improve significantly until effective targeted and tumor-specific agents are developed. Recent advances in targeted therapies provide fresh alternatives for therapeutic development against RMS. Many new and novel agents targeting receptor tyrosine kinases are in various stages of clinical development that may benefit RMS patients, including those targeting PDGFR, EGFR, VEGFR1-3, SRC, and IGF1R [15]. Unfortunately, the inhibition of a single receptor tyrosine kinase only has modest activity in some cases. Additional targeted agents are clearly needed to have better control of the disease.

Apoptosis or programmed cell death is a naturally occurring process for removing unwanted cells in the body. Impaired apoptosis plays a key role in cancer pathogenesis through uncontrolled cell growth and contributes to poor chemotherapy responses. Apoptosis can be achieved by the activation of the intrinsic, mitochondria-dependent pathway or the extrinsic, death receptor-mediated pathway. The frequent inactivation of p53 enables cancer cells not only to bypass the intrinsic apoptotic response to their genomic aberrations, but also to escape apoptosis induced by various conventional DNA-damage therapeutic agents [16]. Therefore, targeting the extrinsic, death receptor-mediated pathway provides a new alternative to current cancer therapies [17].

TNF-related apoptosis-inducing ligand (TRAIL) is a membrane of the TNF family of cytokines [18]. Binding of TRAIL to death receptors DR4 (TRAIL-R1) and/or DR5 (TRAIL-R2) results in the assembly of the death-induced signaling complex (DISC) involving the FAS-associated death domain (FADD) protein and caspase-8 or -10 [19, 20]. Due to the selectivity of TRAIL towards cancer cells, there has been a significant interest in developing agents targeting TRAIL receptors for the treatment of various cancers [17, 21]. Some of them, including the recombinant TRAIL ligand as well as agonistic therapeutic antibodies directed against DR4 and DR5, are currently under clinical development. In this paper, we will discuss the therapeutic potentials of agents targeting the death receptor DR5 for RMS.

## 2. Inducing Extrinsic Cell Death in Tumors via Death Receptor Activation

**Figure 1:**
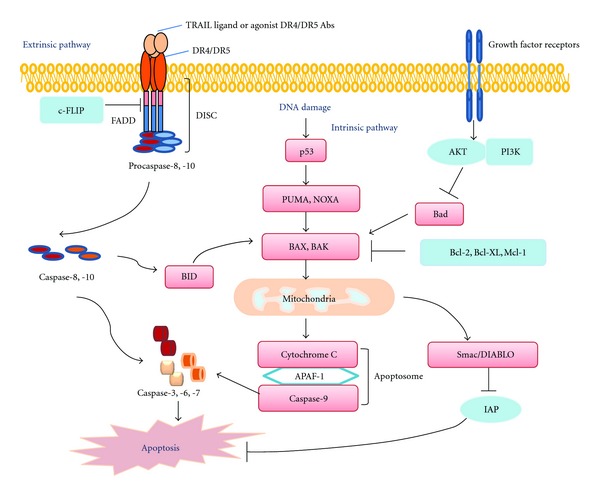
Diagram of intrinsic and extrinsic apoptosis.

A main mechanism for cell death, apoptosis is a natural cellular suicide program aimed to eliminate those cells that are no longer in need or that have sustained severe damage to their DNA [22]. Apoptosis has critical roles in embryonic development and tissue homeostasis. Deregulation of apoptosis is crucial for the development of cancer [23, 24]. The inactivation of the tumor suppressor *p53* enables cancer cells to bypass programed cell death in response to DNA mutations and chromosome aberrations [16]. Apoptosis occurs primarily via intrinsic and extrinsic pathways that are generally separate but sometimes intersect (Figure 1).

### 2.1. The Intrinsic Apoptosis Pathway

The intrinsic pathway is activated by the loss of growth factor signals or by severe cellular stress such as DNA damage and is controlled by members of the Bcl-2 protein family [25, 26]. Activation of proapoptotic family members BAX and BAK results in the permeabilization of mitochondrial membranes, releasing cytochrome C and Smac/DIABLO into the cytoplasm (Figure 1) [27]. The released cytochrome C facilitates the formation of the apoptosome, consisting of Apaf-1, cytochrome C, and procaspase-9. The subsequent activation of caspase-9 leads to the cleavage of downstream effector caspases-3, -6 and -7 [28]. Smac/DIABLO enhances apoptosis by interacting with and blocking the activities of the inhibitors of apoptosis proteins (IAPs) [29].

This pathway is particularly important for cancer therapy since both chemo- and radiation therapies result in DNA damage and the activation of the p53 checkpoint [30]. p53 is a master regulator of apoptosis that responds to a variety of cellular stresses, including DNA damage, hypoxia, and nutrient deprivation [31, 32]. It promotes apoptosis by inducing the expression of proapoptotic genes, including *PUMA*, *NOXA*, *BID*, *BAX*, and *APAF-1* [33]. Various studies have shown that inactivation of *BAX* or *PUMA,* or the overexpression of *BCL-2* or *BCL-xL*, can effectively promote tumorigenesis, suggesting that p53-mediated apoptosis is a significant contributor to tumor development [33].

Notably, RMS is the most common cancer in pediatric patients carrying germline *p53* mutations [34], and mutated *p53* is frequently found in RMS [13, 35]. p53 was shown to mediate radiation and anticancer agent-induced cellular apoptosis [36, 37]. Similarly, p53 is important for conferring cellular sensitivity to chemotherapeutic agents in RMS [38]. Thus, there is a need to explore agents targeting RMS independent of *p53* mutation status.

### 2.2. The Extrinsic Apoptosis Pathway

The extrinsic pathway is activated by proapoptotic receptors on the cell surface. The biological process of the extrinsic apoptosis pathway has been extensively investigated. The binding of TRAIL to the death receptors DR4 and/or DR5 causes the trimerization of the receptors and the recruitment of the FADD protein [19]. 

Subsequently, FADD attracts initiator caspase-8 or -10 through its death effector domain to form the death-inducing signal complex (DISC), in which the initiator caspases are activated by proteolysis (Figure 1). Activated caspase-8 or -10 then cleaves the effector caspase-3, which in turn leads to the cleavage of death substrates. The activation of caspase-8 can be regulated by FLICE-like inhibitor protein c-FLIP [39] and by caspase-8 ubiquitination [40].

There are two types of intracellular signaling linked to the extrinsic apoptosis pathway [50, 51]. In type I signaling, caspase-8 activation is sufficient to commit a cell to apoptosis. The activated caspase-8 or -10 then cleaves downstream effector caspase-3, which in turn results in the cleavage of death substrates. In type II apoptotic signaling, further signal amplification is needed and is achieved through caspase-8-mediated cleavage of Bid. Bid then participates in the mitochondrial-dependent intrinsic pathway to enhance apoptotic activity.

### 2.3. TRAIL Receptors as Therapeutic Targets

Proapoptotic receptors are potentially attractive targets for cancer therapy because they are widely expressed in tumors. In addition, common oncogenes including Myc and Ras, which are often activated in RMS [5, 52, 53], appear to increase tumor sensitivity to the extrinsic pathway [54–56]. More importantly, the extrinsic pathway can be effectively activated regardless of *p53* status, which is useful since loss of p53 function is common in RMS and leads to resistance to traditional chemo- and radiation therapies.

Three ligands belong to the tumor-necrosis factor (TNF) superfamily, TNF*α*, FAS ligand, and TRAIL. The proinflammatory effects of TNF*α* have significantly limited its clinical development [57]. Agonistic antibodies to FAS or FAS ligand were considered unsuitable for clinical development as they cause massive hepatocyte apoptosis and lethal liver damage in animal models [58, 59]. Agents targeting TRAIL receptors DR4 and DR5 were shown to be well tolerated in both preclinical models and phase I clinical trials [41, 46, 60]. Thus, TRAIL death receptors are considered feasible targets for the development of antitumor agents.

Much effort has been made to understand tumor sensitivity and resistance to TRAIL-induced apoptosis. Prior studies showed that DR4 and DR5 receptor levels are not well correlated with sensitivity to apoptosis stimulation. Extensive research was carried out to identify tumor biomarkers predictive of sensitivity or resistance to agents targeting TRAIL receptors. Multiple factors have been suggested to affect TRAIL-induced apoptosis. Decoy receptors DcR1, DcR2, and OPG can bind to TRAIL without mediating death signaling, thereby competing for available TRAIL [61]. The posttranslational O-glycosylation [62] and endocytosis [63] of DR4 and DR5 were implicated as mechanisms affecting TRAIL-induced cell death. Reduced expression of caspase-8 via epigenetic silencing [64, 65] or increased ubiquitination of caspase-8 protein [40] also limits TRAIL signaling. c-FLIP functions as an important inhibitor for TRAIL-induced apoptosis, by competing with the recruitment of caspases-8 and -10 to the DISC [66]. In tumor cells with type II TRAIL-induced apoptosis, cell death can be blocked by the overexpression of antiapoptotic Bcl-2 proteins, such as Bcl-2 and Bcl-xL [17]. Downstream caspase activity can be further inhibited by XIAP [67] and cIAP [68]. Conversely, inhibition of the PI3K/AKT pathway sensitizes tumor cells to TRAIL treatment and reverses TRAIL resistance [69]. These important studies may facilitate the identification and implementation of predictive biomarkers for the clinical development of TRAIL-based therapeutics for cancer.

## 3. Targeting Death Receptors in Rhabdomyosarcoma

### 3.1. Selectivity of TRAIL and DR5 Antibodies to Rhabdomyosarcoma

Amongst various sarcomas, many RMS and Ewing's sarcoma (EWS) cell lines are sensitive to single-agent TRAIL [70–76]. Nearly half of the RMS cell lines examined have a sensitivity comparable to that of the most sensitive breast cancer cell line MDA-MB-231 [70, 76]. The activity of TRAIL appears to be specifically mediated through death receptor DR5 in these RMS cell lines, because only DR5, not DR4, is expressed [76]. This result is further supported by the observation that those RMS cell lines sensitive to TRAIL are also sensitive to antibodies against DR5 but are completely resistant to those directed toward DR4 [76]. Similarly, the majority of Ewing's sarcomas are also sensitive to TRAIL [73]. DR5 receptor also appears to have a greater role in mediating the proapoptotic activity of TRAIL in EWS. Though both DR4 and DR5 are expressed by EWS cell lines, only DR5 is detected on the cell surface [75]. Thus, both RMS and EWS can be effectively induced to undergo apoptosis via the DR5 receptor.

### 3.2. Biomarkers Predictive of TRAIL Sensitivity

Although DR5 is the direct target of TRAIL ligand or its agonistic antibodies in RMS cells, it is expressed by all cells, regardless of their sensitivity to TRAIL or DR5 antibodies. The analysis of the expression of decoy receptors DcR1 and DcR2 did not show a correlation with TRAIL sensitivity in either RMS or EWS [70, 75]. In contrast, investigations revealed a good correlation between the expression of caspase-8 and TRAIL sensitivity in RMS [70, 76]. The level of caspase-8 is clearly important for DR5-mediated apoptosis. In cells with very low caspase-8, the DR5 antibody drozitumab fails to induce the assembly of DISC or the subsequent activation of caspases necessary for apoptosis. More importantly, the expression of wild type, but not catalytically inactive, *CASP8* confers drozitumab sensitivity to resistant RMS cells, indicating that expression of active caspase-8 is a predictive biomarker for RMS sensitivity to DR5-targeted agents [76]. The preclinical analysis of cellular components critical for RMS sensitivity to DR5-targeted agents suggests that caspase-8 should serve as biomarker for subsequent clinical correlative studies.

Caspase-8 is a protein crucial for death receptor-mediated apoptosis. Mutations in *CASP8* were detected in colorectal, liver, and gastric cancer [77–79]. Inactivating mutations were also detected in head and neck tumors [80]. In addition, genomic deletions or silencing of *CASP8* are frequent in neuroblastoma with MYC amplification [81]. While deletion of *CASP8 *is common in neuroblastoma resulting in reduced expression and sensitivity to TRAIL [65, 82, 83], these genomic changes are not common in RMS or EWS [84]. *CASP8 *expression appears to be regulated by hypermethylation, and agents that alter methylation status can lead to increased sensitivity to TRAIL and death receptor activation [83, 85]. It was suggested that the addition of DNA methyltransferase inhibitors, such as 5-dAzaC, may restore *CASP8 *expression and sensitize resistant cells to TRAIL-induced apoptosis in some neuroblastoma and medulloblastoma cells [85]. It is not known whether agents targeting DNA methyltransferase could induce the expression of *CASP8 *in RMS.

### 3.3. Agents Sensitizing Rhabdomyosarcoma and Ewing's Sarcoma to Death-Receptor-Targeted Agents

Like many other targeted agents, the combination of TRAIL-receptor-targeted agents with numerous conventional and investigational anticancer agents has been tested in many preclinical models [21]. The agents used in combination studies include a wide variety of traditional chemotherapeutic agents and radiation, proteasome inhibitors, histone deacetylase inhibitors, and various investigational inhibitors of Bcl-2 and IAP [21]. In EWS cells, the combination of TRAIL with a proteasome inhibitor, a DNA demethylating agent, or interferon *γ* showed initial promise [75, 83, 86]. Combination studies with TRAIL were also performed against RMS cells. In one instance, the chemotherapeutic agent doxorubicin potentiated TRAIL cytotoxicity in resistant RMS cells [87]. Interestingly, other studies revealed that both casein kinases I and II appear to inhibit TRAIL-induced apoptosis in RMSs and shRNA-mediated gene silencing of either kinase increases the sensitivity of RMS to TRAIL [88, 89].

### 3.4. Preclinical *In Vivo* Results with Death-Receptor-Targeted Agents to Combat Rhabdomyosarcoma and Ewing's Sarcoma

Preclinical *in vivo* testing against EWS was performed using lipid-based gene transfer into nude mice inoculated with a sensitive EWS cell line. TRAIL transgene was shown to decrease tumor progression and increase animal survival [71]. Although this gene transfer method is not likely to be implemented in clinical trials, the results suggest that TRAIL can be a promising candidate for therapeutic development. The preclinical investigation of a therapeutic agent against RMS was performed with the DR5 agonistic antibody drozitumab in an SCID mouse xenograft model. The results showed that weekly injection of drozitumab had potent antitumor activity against RMS tumors that was associated with rapid tumor regression and durable response. Further, drozitumab has the selectivity against RMS cells predicted from the *in vitro* cell-based studies [76], thus, providing the preclinical validation of a DR5-targeted agent for followup clinical investigations in patients with RMS.

## 4. Clinical Development of Therapeutics Targeting Death Receptors

There are currently two approaches in targeting death receptors: recombinant human (rh) TRAIL protein that activates both DR4 and DR5 and agonistic monoclonal antibodies that activate either DR4 or DR5 [21]. Recently, both rhTRAIL and agonistic antibodies have entered clinical trials as either single agents or in combination with chemotherapeutic or other targeted agents.

### 4.1. Recombinant Human TRAIL

In a phase I trial in patients with solid tumors or hematological malignancies, rhTRAIL, or dulanermin, was well tolerated [41]. Overall, 46% of patients had stable disease, and two patients with chondrosarcoma had a partial response [41]. One of the potential limitations for dulanermin was its very short half-life between 30 min and 1 hour. Thus, the drug is only at or above effective levels very briefly during the 5-day infusion of each treatment cycle.

Dulanermin was also evaluated in combination with a number of chemo- and other targeted therapies. In metastatic colorectal cancer, dulanermin was evaluated together with irinotecan and cetuximab or FOLFIRI. The initial results suggest that they can be combined safely [90]. Dulanermin in combination with paclitaxel, carboplatin, and bevacizumab has also been evaluated in 24 patients with previously untreated non-small-cell lung cancer (NSCLC) [49]. The combination is safe, and thirteen partial responses and one complete response were reported in this study. The response rate (58%) was considered higher than that in a previous study in advanced NSCLC without the addition of dulanermin (35%). Dulanermin was also evaluated in combination with rituximab in patients with low grade non-Hodgkin lymphoma (NHL) that had relapsed following previous rituximab-containing therapy. Initial results of the five subjects available for analysis revealed that two had a complete response, one had a partial response, and two had stable disease [91]. A larger phase II study comparing rituximab with or without dulanermin in patients with relapsed follicular NHL is in progress. Currently, there is no information or ongoing trials of dulanermin in RMS patients.

As a recombinant TRAIL ligand, dulanermin has a number of unique properties when compared to the agonistic antibodies. It possesses a much shorter serum half-life of 30–60 min [41], less than 1% of that for an agonistic antibody. Thus, dulanermin requires more frequent dosing to achieve a durable antitumor response. In practice, the plasma dulanermin concentration is below the desired level for most of the time during a treatment cycle. Also, dulanermin binds both DR4 and DR5 and could theoretically have a broader activity profile or greater toxicity over agents that are specific for either receptor. It is not clear whether binding to decoy receptors could affect the drug's activity. In addition, unlike many DR4 or DR5 targeted agonistic antibodies, dulanermin does not require exogenous cross-linking or Fc*γ* receptor (Fc*γ*R) interactions for its activity in preclinical studies [92].

Due to the lack of correlation between the levels of TRAIL receptors in tumor cell lines and their drug sensitivities, alternate biomarkers of tumor cell sensitivity are required [93]. Elevated expression of O-glycosylation-initiating and -processing enzymes was correlated with TRAIL sensitivity in various tumor cell lines [62]. Furthermore, DR4 and DR5 were shown to be targets of O-glycosylation that facilitated ligand-induced receptor clustering and caspase activation, suggesting that O-glycosylating enzymes and their targets may be predictive biomarkers of response to the treatment with dulanermin [94].

### 4.2. Agonistic Antibody against DR5

As described earlier, RMS cell lines have neither expression of DR4 nor sensitivity to a DR4 antibody [76]. Thus, only agonistic antibodies against DR5 are likely to be effective. Multiple phase I single agent studies with advanced tumors were completed with lexatumumab [42–44], drozitumab [45], conatumumab [46, 47], and TRA-8/CS-1008 [48] (Table 1). These agonistic antibodies were generally well tolerated at the doses tested, and most did not reach the maximum tolerated dose. These antibodies have a half-life of 1–3 weeks, much longer than that of dulanermin. The antibodies are administered biweekly, far less frequently than the daily dosing with dulanermin. Moreover, due to their long half-lives, the steady-state levels of these antibodies can stay above the effective dose throughout the duration of the treatment, instead of an hour or so during the time of infusion with dulanermin.

Despite multiple reports of stable disease with DR5-targeted agonistic antibodies, of the single-agent trials, only conatumumab was shown to have modest activity at phase I with a partial response in a patient with NSCLC [46]. The initial clinical studies with DR5-targeted antibodies indicate rather low initial clinical activities. Additional work such as antibody optimization, patient stratification based on certain biomarkers, or combination with other agents to synergize the activity of the antibody may be required to achieve better clinical outcomes.

Various agonistic human DR4 and DR5 antibodies display maximal proapoptotic activity in *in vitro* assays upon artificial Fc antibody or protein G cross-linking. In the absence of the cross-linking, most DR5 therapeutic antibodies exhibited low to minimal activity *in vitro* against target tumor cells [95–98]. *In vivo*, their activity requires interaction with the Fc*γ*R that is present on leukocytes [92]. Polymorphisms in human Fc*γ*R affect both antibody binding and the antibody's pro-apoptotic activity against tumor cells [92]. Also, the proximity between target cells and leukocytes expressing Fc*γ*R in solid tumors may constrain the accessibility of the tumors to the cross-linked agonistic antibodies. Our recent results showed that a mouse monoclonal antibody can effectively kill target tumor cells without cross-linking, with a potency comparable to that of the cross-linked drozitumab [76]. Thus, additional optimization of the agonistic antibodies may be required for optimal antitumor activity that is either independent of cross-linking or less subject to the effects of polymorphisms in the human Fc*γ*R.

Combining the death receptor antibodies with standard chemotherapy or targeted agents may enhance their antitumor activities through the crosstalk between the extrinsic and intrinsic apoptotic pathways. Several clinical safety studies of DR5 agonists in combination with chemotherapy and/or targeted agents are in progress in advanced solid tumors (Table 1). DR5 agonistic antibodies can be safely combined with standard doses of cancer therapeutics in small cohorts of patients. The combinations did not lead to significant drug-to-drug interactions or to significantly sensitize normal cells to apoptosis. These combinations include single cytotoxic agents (doxorubicin, gemcitabine, and pemetrexed), cytotoxic agent combinations (carboplatin/paclitaxel, cisplatin/gemcitabine, and FOLFIRI), targeted agents (bortezomib, panitumumab, rituximab, and vorinostat), and cytotoxic-targeted agent combinations (carboplatin/paclitaxel/bevacizumab, FOLFOX/bevacizumab, and irinotecan/cetuximab). A number of such studies of particular interest to RMS researchers and patients include conatumumab (AMG655) and insulin-like growth factor 1 receptor antibody AMG479 in advanced, refractory solid tumors; conatumumab and doxorubicin for the first-line treatment of soft tissue sarcoma. Some of the combination studies have moved to the randomized phase II stage with results anticipated in the next two years.

## 5. Conclusions

Because of the potential promise of inducing programmed cell death independent of *p53* mutation status, agents targeting TRAIL receptor DR4 and DR5 have been evaluated in more than 20 clinical trials as single agents or in combinations [21, 99, 100]. When administered as single agents, they exhibited modest clinical activity with objective responses in two chondrosarcoma patients treated with rhTRAIL [41], and in one NSCLC patient with conatumumab [46]. Until randomized studies are completed, it is too early to tell whether the death receptor targeted agents are active in various combinations. Both dulanermin and agonistic antibodies have limitations that may affect their clinical efficacy. Dulanermin has a half-life of less than one hour and is expected to be effective for a very short period during the 5-day infusion treatment cycle [41]. Most of the agonistic antibodies require cross-linking for their activities *in vitro*. And *in vivo*, their activities may depend on leukocyte penetration and favorable Fc*γ*R polymorphism [92]. Thus, these therapeutic agents targeting death receptors may need to be further improved for stability or cross-linking-independent activity.

As a group, RMS cells show high sensitivity to TRAIL *in vitro*, where TRAIL activity is mediated through DR5 activation [70, 76]. Preclinical studies showed that the DR5 agonistic antibody drozitumab is selective and very effective against a subgroup of RMS both *in vitro* and *in vivo*, with caspase-8 expression predictive of response [76]. The continuous improvement of agents targeting DR5 and patient selection may prove to be critical for the success of this group of agents.

## Figures and Tables

**Table 1:** Death receptor-targeted agents in clinical development.

Agents	Phase	Target Cancer	Current status	Responses	Ref.
Dulanermin	1	Advanced Ca	Completed	2 PR Chondrosarcoma, 46% SD	[41]
Lexatumumab	1	Advanced Ca	Completed	No PR, 32% SD	[42]
Lexatumumab	1	Advanced Ca	Completed	No PR, 29% SD	[43]
Lexatumumab	1	Advanced Ca	Completed	21% SD	[44]
Drozitumab	1	Advanced Ca	Completed	3 minor response, 49% SD	[45]
Drozitumab	2	Chondrosarcoma	Terminated	NA	NA
Conatumumab	1	Advanced Ca	Completed	1 PR NSCLC, 38% SD	[46]
Conatumumab	1	Advanced Ca	Completed	50% SD	[47]
Tigatuzumab	1	Advanced Ca	Completed	41% SD	[48]

				Combination	

Dulanermin	1b	NSCLC	Completed	Paclitaxel, carboplatin, and bevacizumab	[49]
Dulanermin	1b	Colon Ca	Recruiting	Camptosar/Erbitux or FOLFIRI	NA
Dulanermin	1b	Colon Ca	Active	FOLFOX, Bevacizumab	NA
Dulanermin	2	NHL	Completed	Rituximab	NA
Lexatumumab	1	Pediatric Ca	Completed	IFN_*γ*_	NA
Drozitumab	2	NSCLC	Completed	Paclitaxel, carboplatin, and bevacizumab	NA
Drozitumab	1b	Colon Ca	Completed	lrinotecan, cetuximab, and FOLFIRI with bevacizumab	NA
Drozitumab	1b	Colon Ca	Completed	FOLFOX and Bevacizumab	NA
Drozitumab	2	NHL	Completed	Rituximab	NA
Conatumumab	1b	NHL	Suspended	bortezomib or vorinostat	NA
Conatumumab	1b/2	Colon Ca	Active	mFOLFOX6 and Bevacizumab	NA
Conatumumab	1b/2	Advanced Ca	Active	Anti-IGF1R	NA
Conatumumab	1b/2	NSCLC	Completed	Paclitaxel and carboplatin	NA
Conatumumab	1b/2	Soft tissue sarcoma	Active	Doxorubicin	NA
Conatumumab	1b/2	Pancreatic Ca	Active	gemcitabine	NA
Conatumumab	2	Colon Ca	Active	FOLFIRI	NA
Conatumumab	1b/2	Colon Ca	Active	panitumumab	NA

Abbreviations: NHL, non-Hodgkin's lymphoma, NSCLC, non-small-cell lung carcinoma, PR, partial response, and SO, stable disease.
